# GM-CSF - an oncogenic driver of HER2+ breast leptomeningeal metastasis

**DOI:** 10.18632/oncoscience.566

**Published:** 2022-10-10

**Authors:** Rachana Garg, Kai Jandial, Mike Chen

**Keywords:** HER2+ leptomeningeal carcinomatosis (HER2+ LC), granulocyte-macrophage colony-stimulating factor (GM-CSF), tripeptidyl peptidase 1 (TPP1), oligodendrocyte progenitor cells (OPCs), pan-aurora kinase inhibitor (CCT137690)

Leptomeningeal carcinomatosis (LC) is a rare but ominous manifestation of systemic cancer that occurs from the infiltration of cancer cells into the cerebrospinal fluid and leptomeninges. The most common solid tumors exhibiting leptomeningeal spreading include breast, lung, melanoma, gastrointestinal and central nervous system tumors [1]. Within breast cancer, the HER2+ subtype has the most proclivity to form LC. Previous work from our group and others has highlighted that HER2 overexpressing breast tumors show a higher incidence of CNS metastasis and LC than other molecular subtypes [2–4]. Even though the incidence of LC is 5–15% in patients with malignant tumors, it is a significant cause of morbidity and mortality with a mean survival time of 2–6 months. Notably, ~ 1/3 of patients developing LC show no indication of systemic disease. Currently, the gold standard for LC diagnosis includes high-resolution magnetic resonance imaging of the brain and spine followed by a CSF examination. Lately, analysis of CSF tumor markers (VEGF, CYFRA 21-1, and carcinoembryonic antigen), CSF-derived cell-free circulating tumor DNA, and NMR metabolomics have also been considered in the diagnosis of LC. However, despite advancements in diagnostic modalities, LC remains incurable, and there are limited therapeutic options with a considerable risk of treatment-related toxicities. Therefore, it becomes crucial to comprehensively characterize the disease pathogenesis and determine novel therapeutic targets/strategies.

Instituting a standard treatment regimen for LC has been impacted by several factors: heterogenous LC population, rapid progressiveness, low incidence, paucity of adequate randomized clinical trials, and low accrual. The current standard of care for LC management is thus multidisciplinary, including radiotherapy (RT), systemic and intrathecal (ITC) chemotherapies, and surgery [5–8]. Reports have highlighted that the whole brain RT had no survival benefits for LC originating in lung cancer patients. In other incidences, RT has been applied to amend CSF flow by debulking compressive and symptomatic lesions, which enhanced the effectiveness of ITC. Systemic and intrathecal chemotherapies have demonstrated mixed results in improving survival. The blood-brain barrier plays a significant role in constraining conventional chemotherapy and targeted therapies for treating LC. Methotrexate, cytarabine, thiotepa, and liposomal cytarabine are some commonly employed in ITC. However, the major hurdles concerning ITC relate to the delivery, which relies on CSF circulation and challenges arising from toxicity, including septic/chemical meningitis, infectious meningitis, seizure, myelosuppression, and leukoencephalopathy. Yet another recently explored approach for LC management is targeted therapy. These include biomarker-driven therapies, biosimilars, and antibody-drug conjugates; however, extensive prospective studies are needed before considering these regimens into the treatment plan. Bevacizumab (VEGF inhibitor) and dabrafenib (BRAF inhibitor), and anaplastic lymphoma kinase (ALK) inhibitors have shown effectiveness in LC derived from melanoma and lung cancers, respectively. Although substantial progress has been made in alleviating breast cancer, the efficacy of those treatment regimens in the CNS remains challenging; HER2+ LC usually emerges when the systemic tumor burden is well-controlled. Indeed, 30% of the HER2+LC cases are identified as the first manifestation of cancer after a considerable disease-free period has elapsed. Thus far, there are limited options for treating HER2+ LC, and there exists an urgent need to identify novel therapeutic targets to improve the management of this threatening disease. Our current understanding of the molecular mechanisms driving breast cancer metastasis to the brain remains in infancy. In addition, it remains unclear what supports the proliferation of HER2+ breast cancer cells in the acellular, protein, and cytokine-poor leptomeningeal environment.

Our recent comprehensive analysis has defined the signaling events contributing to HER2+ LC development and the pathways that allow its expansion to leptomeninges. To ascertain the role of host glial cells in regulating the growth and development of HER2+ LC, we successfully generated the first-ever expandable primary cell lines from the HER2+ LC patient-derived tumors (“Lepto” cells) [4]. Interestingly, oligodendrocyte progenitor cells (OPC), which occur abundantly in white matter, modulate and hinder the growth of HER2+ LC *in vitro* and *in vivo*, thus restricting the spread of HER2+ LC beyond the leptomeningeal surface. Furthermore, cytokine-array-based analysis conducted on conditioned media derived from the various CNS cell types and Lepto cells revealed higher granulocyte-macrophage colony-stimulating factor (GM-CSF) expression in Lepto vs. other CNS cells. Notably, Lepto-bearing mouse brain sections also exhibited elevated levels of the phospho-GM-CSF receptor α.

The presence of OPCs significantly reduced the HER2+ LC cell viability, as evident from the enhanced apoptotic signal observed in Lepto cells grown in OPC-conditioned hCSF or hCSF containing anti-GM-CSF antibodies than that in naive Lepto cells. Reduced activation of GM-CSFR α and downstream targets STAT5, AKT, and ERK1/2 further corroborated the essential role of OPC in regulating GM-CSF secretion from the HER2+ LC cells. LC/MS-MS-based analysis of secretomes of human astrocytes and OPCs, cultured alone or with Lepto cells, revealed TPP1 as the regulator of GM-CSF; it mediates GM-CSF proteolytic degradation and suppresses signaling, thereby decreasing Lepto cell viability and tumor progression. Remarkably, combinatorial treatment of anti-GM-CSF antibody with a pan-Aurora kinase inhibitor (CCT137690) synergistically halts GM-CSF signaling and HER2+ LC growth *in vitro* and *in vivo*.

**Figure 1:**
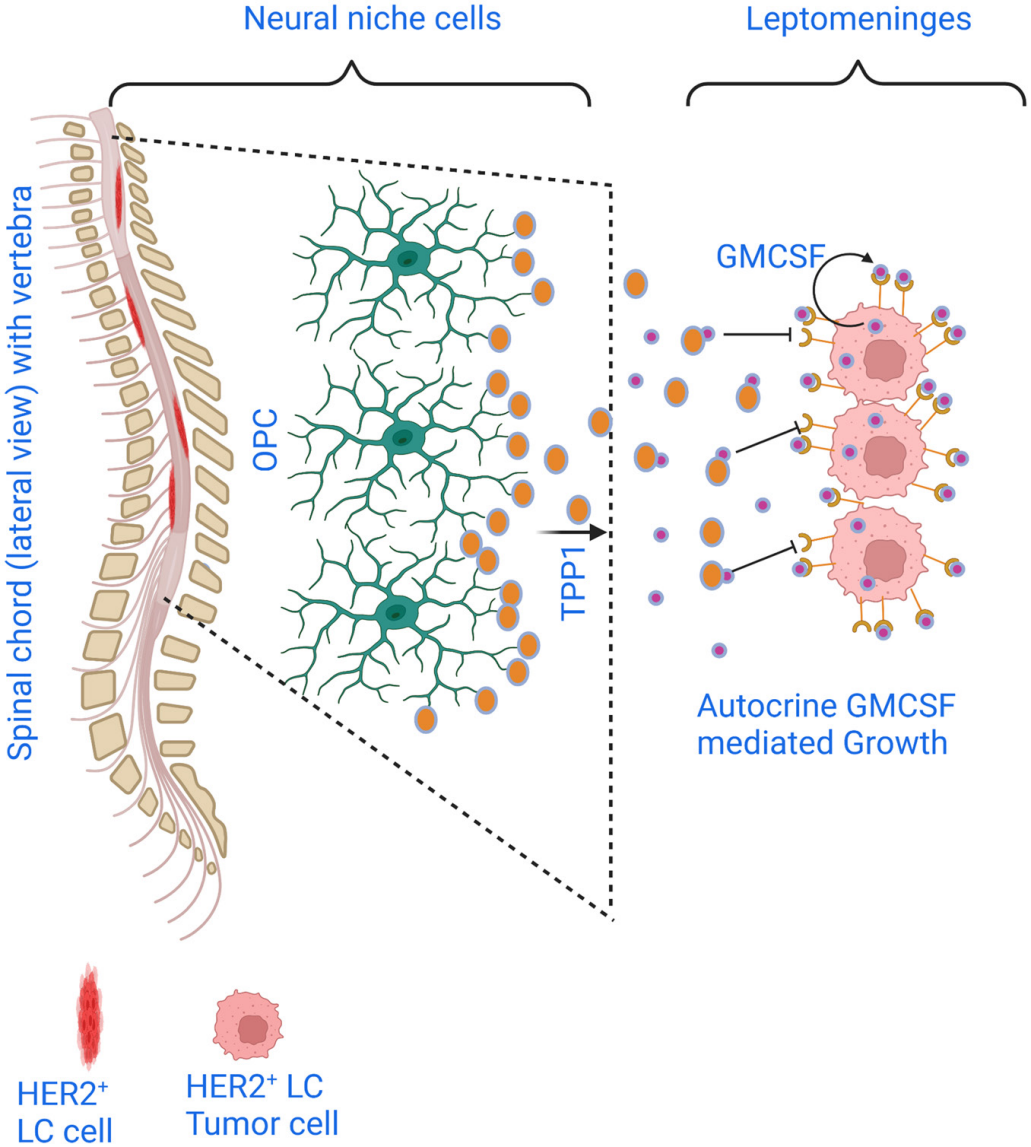
Crosstalk between breast cancer cell GM-CSF and OPC-secreted TPP1 in the leptomeningeal environment modulates the growth of HER2+LC.

Our work has demonstrated neural niche-specific crosstalk between HER2+ LC tumors and OPCs and established the efficacy of intrathecal administration of TPP1 protease, anti-GM-CSF antibodies, and pan-Aurora kinase inhibitors in combating HER2+ LC (Figure 1). This may offer a targetable axis to treat HER2+ LC in the clinics.
